# Conducting dyadic, relational research about endometriosis: A reflexive account of methods, ethics and data analysis

**DOI:** 10.1177/1363459318786539

**Published:** 2018-07-06

**Authors:** Nicky Hudson, Caroline Law, Lorraine Culley, Helene Mitchell, Elaine Denny, Nick Raine-Fenning

**Affiliations:** De Montfort University, UK; Birmingham City University, UK; Nurture Fertility, East Midlands Fertility Centre, UK; University of Nottingham, UK

**Keywords:** chronic illness and disability, couples, dyadic methods, endometriosis, experiencing illness and narratives, issues in research methodology, qualitative research, research methodology

## Abstract

Despite a growing literature on the value of relational data in studies of social phenomena, individuals still commonly constitute the basic unit of analysis in qualitative research. Methodological aspects of interviewing couples, particularly interviewing partners *separately*, and of conducting dyadic analysis have received scant attention. This article describes the experience of conducting separate interviews with both partners in 22 heterosexual couples (n = 44) in a study of the impact of the gynaecological condition endometriosis. In order to advance current methodological thinking regarding interviewing couples, we describe the dyadic, relational approach employed in designing the study and our specific method of dyadic analysis. We argue that utilising separate interviews with dyadic analysis rather than conducting joint interviews, while not without its ethical, practical and analytical challenges, offers considerable methodological benefits. Such an approach allows a unique relational insight into the impact of chronic illness on couples and how they navigate chronic illness by illuminating both shared and individual interpretations, experiences, understandings and meanings.

## Introduction

Despite a growing literature on the value of relational data in studies of social phenomena (May, 2011; Smart, 2007), individuals still commonly constitute the basic unit of analysis in qualitative research. Studies of illness experiences commonly give primacy to the account of the person living with the condition, and while this allows a dedicated focus on personal, subjective accounts, in some cases it neglects an important opportunity to explore the relational nature of health and the specific ways in which social networks and intimate relationships can configure lives and experiences. Interviewing couples can offer rich relational data, which may be especially relevant in research about chronic illness because of the potential impact of illness on relationships with family members and partners and because of partners’ roles in managing illness and coping day to day (Culley et al., 2017; Hudson et al., 2016).

Relationality, and the ‘relational turn’ in sociology, is concerned with understanding the constitution of a phenomenon through figurations, networks or social worlds and sees social relations as dynamic and fluid processes (Dépelteau and Powell, 2013; Finch, 2007; Roseneil and Ketokivi, 2016; Smart, 2007). A relational framing therefore requires the use of methods that are able to effectively explore the points of connection between social actors (Hudson et al., 2016; Springer et al., 2012). There exists a long tradition in the sociology of the family of interviewing multiple family members which has directly informed the use and development of relational methods more generally (Finch, 2007; Roseneil and Ketokivi, 2016; Smart, 2007; Valentine, 1999). However, the literature remains incomplete and inconclusive with regard to the question of whether interviewing jointly or separately is most desirable, with notably fewer authors addressing the latter approach. Furthermore, few authors have tackled the specific complexities inherent in analysing data from couple interviews. The development of relevant methods and approaches is therefore needed in order to further the advancement of a relational sociology of health and illness more generally.

This article offers methodological reflections arising from a study of the impact of the gynaecological condition endometriosis on couples (findings from the empirical data are reported elsewhere; see Culley et al., 2013a, 2017 and Hudson et al., 2016). It describes our rationale for interviewing partners separately, as well the related implications, benefits and challenges. This article seeks to contribute to the small body of methodological literature on interviewing couples and on dyadic analysis and to offer insights to inform future relational and dyadic research. Despite ethical and interpretive complexities, it is argued that the approach we employed presents a number of advantages. We begin with an outline of the study on which this article is based, which is followed by the rationale for the methodological approach we adopted and the outcomes it achieved. The second half of this article describes our approach to undertaking dyadic analysis, as well as challenges associated with reporting the data derived from these methods. We use examples throughout to illustrate the challenges and advantages presented by our approach.

## The ENDOPART study and our methodological approach

Endometriosis is a common gynaecological condition, which occurs when endometrium (the lining of the womb) grows outside the womb (De Nardi and Ferrari, 2011; Dunselman et al., 2014). The main symptoms of endometriosis are pelvic pain, heavy and painful periods, fatigue, dyspareunia (pain during sex) and subfertility (De Nardi and Ferrari, 2011; Lemaire, 2004; Meuleman et al., 2009). There is no definitive cure, but there are several treatments aimed at suppression of the disease and symptom relief with varying degrees of success (Dunselman et al., 2014).

Endometriosis has a significant impact on the lives of women, and while research suggests that couple relationships are affected (Denny and Mann, 2007; Seear, 2009), few studies have included this as a specific focus or have included women’s partners (Culley et al., 2013b). The ENDOPART study was designed to explore the experience of heterosexual couples living with endometriosis and was conceptualised from a relational, dyadic perspective. Using a qualitative methodology, it aimed to explore the impact of endometriosis on women and their male partners, contribute to the development of theory in chronic illness and contribute to improving the wellbeing of people living with endometriosis by providing an evidence base for improving couple support. It comprised three main phases: first, a ‘context-setting’ phase, comprising interviews with key informants and a systematic literature review (Culley et al., 2013b); second, in-depth, semi-structured interviews with women with endometriosis and their male partners; and finally, a stakeholder workshop was held to inform the recommendations and outcomes from the study.

A total of 22 couples were recruited to phase 2, and partners were interviewed separately (n = 44). Couples were recruited via the national charity, Endometriosis UK (n = 11), NHS clinics (n = 5), other support or information groups or organisations (n = 3) and word of mouth (n = 3). Participants were given written information about the study and provided written consent. Interview schedules for the women and their male partners were developed dyadically, for example, comprising similarly themed questions as well as a sub-set of questions to allow for direct comparison of perspectives (see further details below). Interviews were recorded, transcribed verbatim and entered into NVivo for analysis. Ethical approval was granted by the host university and by the East Midlands Leicester NHS Local Research Ethics Committee UK (reference 12/EM/0015).

In designing the study, the decision was made to use individual interviews but to design and analyse them according to a dyadic approach. We argue that dyadic research can be defined as that which takes a dyad – that is two people in a pre-existing relationship – as the unit of study and foregrounds this dyadic relationship in the study design and analysis (Eisikovits and Koren, 2010; Morgan et al., 2013; Ummel and Achille, 2016). In dyadic studies, the study aims, design and analytic focus treat relational aspects (e.g. social ties, networks, interactions, processes, etc.; Crossley, 2015) as the focus of inquiry, investigating accounts dialogically and looking at partners’ meanings in dialogue with one another. In keeping with previous literature, we argue that a dyadic approach does not necessitate joint interviews, but that dyadic analysis can be undertaken on separate interviews with partners in a dyadic unit, with this approach comprising one of several possible ‘dyadic data collection modalities’ (Eisikovits and Koren, 2010: 1643).

At the stage of study design, we purposefully adopted a two-part model of ‘methodological best practice’ in order to establish rigour during interviewing. Within this model, we determined that where it was feasible (1) participants would be interviewed separately and (2) interviews would occur simultaneously, and therefore, a different interviewer would be used for each partner. While we aimed to adhere to this model for each couple, this was not always possible due to restrictions in participant availability (e.g. relating to childcare), researcher availability or funding for travelling long distances (the instances in which we were not able to adhere to this model and the reasons for this are discussed below). This was a pragmatic approach which acknowledged that it may not always be possible to follow best practice, but that where logistically possible we could ensure a robust and repeatable data collection process within the resources and timescale of a publicly funded research project.

In the following section, we discuss the rationale, implications, challenges and benefits associated with these two aspects of the model: interviewing partners separately and interviewing partners simultaneously with different interviewers. Following this, the second half of this article describes our approach to undertaking dyadic analysis, as well as challenges associated with reporting.

### Interviewing partners separately

While separate and joint interviews each have relative advantages, family researchers have often advocated separate interviews when researching couple-sensitive topics (e.g. sex and intimacy) and when discussing dynamics and power relationships (Valentine, 1999). Interviewing partners separately enables each participant to ‘tell the story from his or her own perspective, without having to consider the reaction of the other when voicing criticism or bringing up sensitive topics’ (Eisikovits and Koren, 2010: 1643–1644; see also Morris, 2001; Ummel and Achille, 2016). Using this approach increases the likelihood each interviewee will disclose information that they would be unwilling to share in a couple interview (Valentine, 1999) and potentially reduces the related ‘social desirability effect’ that would come into play if their partner was present (Taylor and De Vocht, 2011).

In an insightful piece of work, specifically exploring the gendered nature of joint versus individual interviewing, Seale et al. (2008) compared data from joint and solo interviews in studies about health, pregnancy and parenting experiences. They found that in joint interviews, women were ‘more likely to achieve quantitative dominance’ and suggest that this may be because they were considered, by both their partner and the interviewer, to be the more appropriate spokesperson on these topics (Seale et al., 2008: 124). The experience of living with or alongside endometriosis is, of course, focused on the health and wellbeing of the affected woman. It follows therefore that many aspects of couples’ everyday lives are constructed around the impact of endometriosis on the female partner. In planning our study, we felt that this would also very likely have affected the interview dynamics, since in most cases the female participants would likely (and justifiably) have dominated the interviews. Seale and colleagues therefore advise that if researchers want to find out more about men’s experiences, they should strive to design their research, and specifically their interview questions, in a way that focuses on men’s lives and experiences (Seale et al., 2008).

Following the rationale in these studies – that individual interviews afford more space to discuss sensitive issues and that women living with the condition may have achieved ‘quantitative dominance’ – we interviewed all 44 participants in our study in separate, individual interviews. This approach afforded us a range of benefits in line with our specific objectives of wanting to address the absence of men in previous studies on endometriosis and importantly, to hear men’s accounts separately from those of their female partners. Data from our interviews confirmed this decision, since participants expressed feelings and experiences unlikely to have been forthcoming in a joint interview (Valentine, 1999). For example, both female and male partners reported concealing some aspects of living with endometriosis from their partners. In the following excerpt, one woman talks about how she hides the pain she experiences during sex from her partner:

*I’d love to be one of these couples who put everything on the table and say ‘this is how it is, this and this and this’ but it’s just not how we work. If I told him, if I put everything out and said ‘when we have sex this is how I feel’, he’d run a mile, he would never want to touch me again.* (Female partner)

In the following example, this male partner discusses how he chooses not to reveal to his partner his concerns about whether or not they will be able to have children as a result of her endometriosis:^1^

Male partner: *‘It’s a worry to me that we might not be able to have children’*.

Interviewer: *‘Have you talked to [partner] about your worry?’*

Male partner: *‘No probably not, not as much as, it’s something I am not as open with because, I don’t know, I don’t want her to feel like it’s her fault because it’s not. I think if I just said that I was really worried and the worries that I do have might make her feel bad and I don’t want that … it’s probably something I have kept more concealed just for the sake of sparing her feelings’*.

In discussing the impact endometriosis had on them, men frequently positioned women’s needs as paramount and as a result some felt that expressing their own emotions, concerns or needs within the relationship would be inappropriate or selfish. Separate interviews therefore allowed men to express their own views and feelings, whereas joint interviews may have resulted in a paucity of such data. Furthermore, the interviews highlighted that men’s needs in relation to living alongside endometriosis may be marginalised as the focus of support and treatment resides with the female partner (Culley et al., 2017). This marginalisation may have been further exacerbated with the use of joint interviews:

*I’m so glad you’re asking because … like I said before the chaps just get, you know, with doctors, nurses, everything going on, and they’re just so worried about their wives, girlfriends and they’re just pushed to one side and it’s so important to get their point of view.* (Male partner)

Interviewing partners separately also potentially reduces the related ‘social desirability effect’ that would come into play if their partner was present, that is, the tendency for interviewees to perform or present a particular ‘self’ deemed acceptable to their partner (Taylor and De Vocht, 2011; Werner et al., 2004). However, while this tendency is reduced in separate interviews, we found that social desirability effects do not disappear completely and that this can include the way in which an interviewee presents both themselves and their partner to the interviewer (Taylor and De Vocht, 2011). As Eisikovits and Koren (2010) have suggested, even when separate interviews are conducted, there exists ‘a joint relationship and history, and as a result the partner is often virtually present in the interview space’ (p. 1644).

In our study, this manifested in participants’ desire to portray their partner and relationship in a positive light, and this was done by both men and women albeit in slightly different ways. Men appeared defensive and protective of their partner especially when talking about the legitimacy of their symptoms and their partners’ experiences with unsupportive healthcare practitioners, employees, colleagues and friends and family. Women in comparison were more willing to speak negatively about their partner but when they did, were likely to include qualifying statements about how hard their partner was trying or how supportive they were and so repositioning them in a positive light. Many appeared to avoid or minimise speaking in a way that could be perceived as a betrayal and to avoid being judged as a ‘bad or unmatched couple’ (Valentine, 1999: 71). Nonetheless, alongside these displays, participants also spoke at length about negative aspects of their relationship and in many cases did voice criticism of their partner; therefore, while displays of social desirability were evident, these did not dominate the interview and may have been more frequent in joint interviews.

Separate interviews have been criticised for missing the opportunity to capture interaction and observe negotiation, mediation and dominance between partners within an interview (Arksey, 1996; Morris, 2001; Seale et al., 2008; Valentine, 1999). However, we would suggest that any *observational* data gathered in joint interviews are substantively different in nature from the *verbal* accounts provided by interviewees and that this may therefore present challenges in analysis; something which is seldom explored. As Morgan (2010) argues, treating interaction as something which produces data is different from treating interaction *as* data. This study employed a constructivist and relativist philosophical position, avoiding the pursuit of an underlying ‘truth’ (see further discussion below). In keeping with this position, we argue that interview accounts should be seen as social constructions, subject to a range of shifting positionalities and contextual factors, and that joint accounts are not therefore any more ‘valid’ than separate ones.

### Interviewing partners simultaneously with different interviewers

A second feature of our methodological model was that different interviewers interview partners within a couple unit simultaneously where possible, allowing for the logistical restrictions described above. Interviewing partners in this way avoids the possibility of partners discussing issues with each other between interviews and thus prevents the possibility that an interview with the second partner is influenced by such a discussion (Eisikovits and Koren, 2010; Ummel and Achille, 2016). As suggested above, while we are not assuming in epistemological terms that accounts can ever be ‘untainted’ or unbiased, nevertheless we wanted, where feasible, to give participants the opportunity to tell their story uninhibited by prior expectations or discussions about the interview, or by their partner’s experience of taking part.

As stated above, we were not always able to adhere to this model for practical reasons. In practice, we achieved this intention in 17 out of 22 cases. In the remaining 5 cases, this was not possible due to participants’ childcare requirements and/or researcher availability or travel resources. However, in 4 of these cases, the interviews were conducted in immediate succession by the same interviewer. In one final case, we were unable to time the interviews to happen simultaneously or in immediate succession, and instead, they took place 4 ;days apart. Therefore, we achieved our objective of ensuring that couples could take part without prior discussion of a partner’s interview in 21 out of 22 cases.

This approach had very practical implications. Using two researchers increases the time, cost and organisation needed for data collection. In most cases, we were required to co-ordinate four diaries (two researchers and both members of the couple). This approach also doubles the travel (and if needed, accommodation) costs for the research, something which needs to be factored into funding applications for dyadic research. There were also practical issues for some couples who wanted to do the interviews at home and were therefore required to find two suitable rooms in the house for interviews to take place. This was not always possible and so in some cases led to the need for an alternative location for the second interview. For those with children, booking the interviews at an appropriate time where they could both take part raised childcare issues. Finally, in some cases, the logistics made it just too difficult and/or expensive for two researchers to attend; for example, in two cases, the distance to the couple was so great that to send two researchers was not possible. One interviewer attended and conducted both set of interviews sequentially. This adds further challenges for the researcher when interviews are lengthy and sensitive.

The decision to use different interviewers in simultaneous interviews was also informed by a desire to reduce the possibility of the interviewer bringing prior knowledge, gained in the first interview and relating to the couple unit, into the subsequent interview (Eisikovits and Koren, 2010; Ummel and Achille, 2016). Doing so raises ethical concerns and specifically presents potential difficulties for confidentiality if the interviewer inadvertently discloses information previously given by their partner (Tolich, 2002; Zarhin, 2018). There is also the possibility that interviewees directly ask the interviewer to tell them what their partner said or that the interviewer is asked to take sides (Zarhin, 2018). Indeed, this was raised by one participant, despite the interviews taking place simultaneously:

Female partner: *‘I wonder what sort of questions your friend’s going to ask him. Tell me’*.

Interviewer: *‘Quite similar questions really, it’s quite conversational’*.

Female partner: *‘Will she ask him questions like “how does your wife cope with the housework?” and things like that or would she ask him questions just about him? … Tell me what he thinks. He won’t mind’*.

Participants may also have felt reassured and more comfortable expressing themselves knowing that their interviewer would not, following their interview, be then also meeting with their partner:

Male partner: *‘[Partner] will, we’ll be sitting there on the sofa watching the telly – [partner] doesn’t get to hear this does she?’*

Interviewer: *‘No absolutely not’*.

Male partner: *‘There are certain little things that she does that I’m aware of and I’ll say ‘oh do you need a tab [tablet]?’, and she’ll say ‘how did you [know]?’*

Using different interviewers therefore avoids a situation of a researcher being ‘stuck in the middle’ of a couple and the ethical implications this presents (Forbat and Henderson, 2003).

## Dyadic analysis and reporting: our approach, benefits and challenges

While there is now a small but growing literature on the relative benefits of separate or joint interviews for the data collection process, much less has been written about the process of analysis. In dyadic research, the couple is the unit of analysis; it is not enough to sample both partners – it is the focus on relationships and patterns within couple units that makes research dyadic – the ‘we’ of the experience (Eisikovits and Koren, 2010). Eisikovits and Koren (2010) appear to offer the most in terms of outlining a detailed and systematic dyadic analytic method. They suggest that dyadic analysis alters the individually based interpretation of the data and gives an additional dimension to understanding. Epistemologically they suggest that the researcher creates a third dyadic version, while retaining the individual accounts, highlighting that ‘the dyadic version is more than the sum of two individual ones’ (Eisikovits and Koren, 2010: 1652). In order to achieve this, they propose the following strategy. First, analysis of the individual interviews in line with usual principles of qualitative analysis, that is, a thematic ‘horizontal’ analysis, which seeks to construct a number of common themes across the cases. In the second, dyadic stage, the focus is on each couple unit and comprises a systematic identification of the contrasts and overlaps between partners’ accounts. This is carried out at two levels: the ‘textual and descriptive’ level, which they suggest reveals what they term the ‘open reality’ of the situation, and the ‘sub-textual and interpretive’ level, which seeks to explore what they term the ‘hidden reality’ in the accounts, that is, how the individuals are interpreting phenomena (Eisikovits and Koren, 2010: 1653). In the ENDOPART study, we adapted Eisikovits and Koren’s model, as described below.

### Our approach to dyadic analysis

Our approach to dyadic analysis began at the level of question design (Eisikovits and Koren, 2010). As well as employing a general dyadic method to questioning (which involved asking female and male participants similarly themed questions), we also devised a sub-set of interview questions that were designed to allow us a more direct comparison of perspectives in the analysis. For example, we asked participants ‘*what is the single biggest issue for you and what do you think is the single biggest issue for your partner?*’ which allowed us to explore the similarities and differences in the direct responses to this question. While we recognise the limitations of such directive questions, this strategy provided some focused data on the relative agreement within the couple about the global impact of endometriosis on their lives as well as participants’ levels of awareness regarding their partner’s experience, when compared to the more open data collected about a number of more specific domains such as home life, work, childcare, healthcare and fertility (see Culley et al., 2013a, 2017 and Hudson et al., 2016 for findings).

Second, we used the themes that emerged from the stage 1 thematic analysis to directly inform our stage 2 dyadic analyses. While Eisikovits and Koren suggest that dyadic analysis is conducted throughout the entire data set, our stage 1 data analysis demonstrated that certain themes were more dyadic in character than others and that a more detailed, intense focus on these themes would provide a richer relational account of the couple experience of endometriosis (see Hudson et al., 2016, for discussion of these findings). Two themes were selected according to their dyadic nature: that is how significant they were for the ‘we’ relationship. They were ‘sex and intimacy’ and ‘planning for and having children’. In contrast to research where the questions are centred exclusively on the substantive matter of couple *relationships* (as in Eisikovits and Koren’s (2010) study), in our study, we were also interested in gendered individual experiences of living with/alongside a specific chronic condition and therefore we also sought to retain an empirical focus on women’s and men’s individual accounts as well as exploring the impact at the couple level. This decision was therefore led by our specific study objectives, but means that only specific elements of our data were analysed dyadically and as such our approach signifies a slight departure from Eisikovits and Koren’s approach.

Eisikovits and Koren’s model focuses on the identification of contrasts (opposing descriptions of situations, phenomenon, feelings and experiences) and overlaps (converging descriptions of situations, phenomenon, feelings and experiences) in partners’ accounts, and we followed this approach for our two dyadic themes. A further adaption we made to their model was the identification of ‘*omissions*’ as a particular kind of contrast in partners’ accounts, and one which we suggest represents a new analytical category in dyadic research. Omissions were represented by topics that were discussed by one partner but not the other. This allowed us to further interrogate the different emphasis each partner gave to particular issues. For example, with regard to sex and intimacy, analysis revealed that men frequently omitted several topics that their partners discussed, including bleeding during or after sex, seeking advice and support externally and the loss of intimacy. With regard to planning for and having children, men frequently omitted discussion about biographical disruption, the challenges in negotiating fertility-compromising pain treatments and trying to conceive, and a lack of support or pressure from families, whereas women were more likely to omit discussions about the financial implications (e.g. of in vitro fertilisation (IVF)) and either their or their partners’ coping strategies. These were not identified at the general level, that is, by comparing the data set from all men with the data set from all women, but at the *couple level* by identifying contrasts, overlaps and omissions relating to these factors between partners’ accounts. Exploring the degree of completeness in accounts (Van Dijk, 2001) illuminates the ways in which participants’ narratives are partial. The identification of what is omitted is insightful in understanding the factors that do and do not constitute individuals’ social worlds: ‘what is left unsaid is often more important that what is said’ (Huckin, 2002: 162). In a dyadic study, this is of paramount importance: comparing partners’ accounts in this way allows for an exploration of the factors which do and do not constitute each individual’s experience of endometriosis and how these might differ between partners within a couple unit. Furthermore, including the analytic category ‘omission’ was necessary in order to avoid an incomplete coding exercise. It became apparent early on in the activity that if the data could only be coded to ‘contrast’ or ‘overlap’, this would have left a considerable amount of data uncoded.

Finally, as described above, Eisikovits and Koren (2010) propose that stage 2 of the analysis should involve the exploration of contrasts and overlaps at two different levels: the ‘textual and descriptive’ and the ‘sub-textual and interpretive’ level (p. 1653). We opted not to systematically apply the two levels of analysis in our study for two reasons. This was partly practical, due to the size of our sample (n = 22 couples, 44 interviews) and the resource implications of focusing in such a detailed way on a data set of this size, but mainly this related to the ontological and epistemological tensions that this approach presents (Bjørnholt and Farstad, 2014; Manning and Kunkel, 2015). Seeking to explore the interpretive or ‘hidden meaning’ in the accounts was not in keeping with the constructivist philosophical position we had adopted in the research more generally (Braybrook et al., 2017). We proposed that the cultural repertoires which people draw upon are important in understanding illness experiences and therefore decided to keep our analysis of overlaps, contrasts and omissions at the textual and descriptive level in order to compare the ways in which couples discussed and presented their experiences and perceptions in the interviews, but not to attempt to ‘go beyond’ these representations in our analysis. While we recognise the existence of a relationship (albeit a complex and contested one) between people’s representations and their experiences (Bjørnholt, 2011), we were not attempting to directly assess or evaluate these ‘real life’ experiences via the couples’ accounts.

Overall, this approach to dyadic analysis – exploring contrasts, overlaps and omissions in partners’ accounts – enabled a detailed insight into the impact of the condition on the couple relationship.^2^ It illuminated a range of shared and different interpretations, experiences, understandings and meanings within each couple unit. These kinds of analyses allow a consideration of how life with or alongside the condition can result in markedly different experiences for each partner and contrasting coping strategies which may cause challenges for couple wellbeing, and can produce and re-produce particular gendered subjectivities.

### Our approach to reporting

Interviewing members of a dyad separately causes complexities in subsequently reporting the data (see, for example, Saunders et al., 2015; Ummel and Achille, 2016). An accepted convention when reporting quotations from interviews is to assign a descriptive label to the quotation, providing the reader with contextual or demographic information about the participant (e.g. individual’s age, length of relationship and time since diagnosis). This has not been possible because identifying the data in this way, especially in a small study, may allow partners to identify one another and thereby know exactly what their partner said in the interview.

An alternative might be to use a unique identifier code or a pseudonym; however, if couple quotations are presented together (e.g. male 1 and female 1) and a participant recognises their own quotation, they will therefore also be able to employ ‘jigsaw identification’ to identify the other quotation as coming from their partner (Forbat and Henderson, 2003; Saunders et al., 2015). If a participant could identify one quotation from their partner, they would then also know that all other quotations assigned to that particular unique identifier or pseudonym were also from their partner.

These issues are all the more significant in a study which included distressing accounts and the potential for this information, if identities were uncovered, to have profound effects on relationships (see also Ummel and Achille, 2016). For example, one partner spoke several times about considering ending their relationship. Although we alerted couples, in the information we provided prior to them giving consent, to the slight possibility that they may identify one another from their quotations, we could not have fully anticipated the extent of the impact of endometriosis on some couples and the potential implications of participants identifying their partner’s accounts. Data management and anonymity therefore became of added importance when reporting findings at conferences and in publications. Because of the challenges associated with descriptive labels, unique identifiers or pseudonyms, and due to the enhanced relational sensitivity of the data, we have used ‘female participant’ and ‘male participant’ to present findings in a way that does not compromise internal confidentiality (Tolich, 2004). In addition, we have limited the extent to which we present accounts from both partners side by side, only doing so when necessary. In these cases, we have also avoided the use of direct quotations and removed or modified specific details deemed to be highly identifiable and/or relationally sensitive, while still seeking to provide a valid illustration of the arguments presented. While this approach may compromise the integrity of the data, and is a difficult balance to strike, ultimately we have prioritised ethical defensibility over richness in reporting dyadic analysis (Saunders et al., 2015; Ummel and Achille, 2016).

## Conclusion

The relational turn in the social sciences requires us to consider more effective ways of understanding the complexity of social life and social relations between individuals. In the context of research about long-term conditions such as endometriosis, this can facilitate an improved understanding of the ways in which individuals, couples and families navigate a complex web of symptoms, diagnoses and medical and non-medical management. While individual, solo interviews offer privileged access to individual narratives about lives shaped by chronic illness, a dyadic approach can enrich research accounts and offer a means by which to understand the wider social and relational implications of health and illness as it impacts on social networks and how these connections in turn may act as a source of support or a source of additional stress.

Despite these potential advantages, there is a paucity of literature on interviewing couples and the specific methodological challenges of this approach, especially in relation to data analysis. There is a particular gap in the methodological literature regarding the unique contribution and advantages offered by interviewing partners in a dyad *separately*. Reflections from this study therefore contribute to a small body of methodological scholarship on interviewing couples and offer novel insights on the practical, methodological and ethical dilemmas involved in conducting separate interviews about chronic illness with both partners in a heterosexual couple. We were able to explore accounts that were unlikely to have emerged in joint interviews, and which validate the decisions made regarding separate, simultaneous interviews. This approach allowed participants to articulate perceptions and experiences considered to be relationally or emotionally highly sensitive and to share problematic aspects of relationships, and permitted men’s accounts to be heard unmediated by women’s participation. The best practice approach employed, coupled with a dyadic analysis employing a careful and detailed focus on partners’ accounts in relation to one another, allowed a unique insight into how couples navigate this common chronic condition.

This approach is not without its challenges, including the display of social desirability ‘talk’ within the interviews, the need to carefully consider logistical and resource implications in planning and managing the project and specific ethical concerns regarding anonymity when reporting. Some of the challenges and limitations of separate interviews may be addressed by combining both joint and separate interviews in the same study (see for example Butt and Chesla, 2007). Such a strategy could offer a way to capture relational accounts while minimising the unique limitations of each approach. However, it may present additional challenges and complexities and its appropriateness will depend on the aims of the study in question (Ummel and Achille, 2016).

Despite the challenges presented, interviewing partners separately and undertaking dyadic analysis offered us an effective method for the exploration of couple experiences of endometriosis. Adopting a model of methodological best practice afforded us some flexibility when negotiating the realities of carrying out funded health research and allowed us to make decisions guided by a pre-agreed protocol, supported by existing research evidence, which was tailored to our specific research objectives. This approach also allowed us the space to systematically record, reflect upon and report the success of our design and its associated limitations and offered us a means to gain detailed insight into the impact of the condition on the couple relationship. We would therefore recommend this approach to others who wish to consider the use of such a method.

## Notes

1. Endometriosis can impact fertility. It is estimated that 47 per cent of infertile women have endometriosis (Meuleman et al., 2009).

2. A more comprehensive and detailed overview of findings has not been provided in this paper, due to its primary focus on methodological reflection, but can be found elsewhere (see Culley et al., 2013a, 2017 and Hudson et al., 2016).
